# A Metagenomic Analysis of Pandemic Influenza A (2009 H1N1) Infection in Patients from North America

**DOI:** 10.1371/journal.pone.0013381

**Published:** 2010-10-18

**Authors:** Alexander L. Greninger, Eunice C. Chen, Taylor Sittler, Alex Scheinerman, Nareg Roubinian, Guixia Yu, Edward Kim, Dylan R. Pillai, Cyril Guyard, Tony Mazzulli, Pavel Isa, Carlos F. Arias, John Hackett, Gerald Schochetman, Steve Miller, Patrick Tang, Charles Y. Chiu

**Affiliations:** 1 Department of Biochemistry and Biophysics, University of California San Francisco, San Francisco, California, United States of America; 2 Department of Laboratory Medicine, University of California San Francisco, San Francisco, California, United States of America; 3 UCSF-Abbott Viral Diagnostics and Discovery Center, University of California San Francisco, San Francisco, California, United States of America; 4 Department of Medicine, University of California San Francisco, San Francisco, California, United States of America; 5 Department of Pathology and Laboratory Medicine, University of British Columbia, Vancouver, British Columbia, Canada; 6 British Columbia Centre for Disease Control and University of British Columbia, Vancouver, British Columbia, Canada; 7 Department of Laboratory Medicine and Pathobiology, University of Toronto, Toronto, Ontario, Canada; 8 Ontario Agency for Health Protection and Promotion, Toronto, Ontario, Canada; 9 Mt. Sinai Hospital, Toronto, Ontario, Canada; 10 Instituto de Biotechnología, Universidad National Autónoma de Mexico, Cuernavaca, Mexico; 11 Abbott Diagnostics, Abbott Park, Illinois, United States of America; University of Georgia, United States of America

## Abstract

Although metagenomics has been previously employed for pathogen discovery, its cost and complexity have prevented its use as a practical front-line diagnostic for unknown infectious diseases. Here we demonstrate the utility of two metagenomics-based strategies, a pan-viral microarray (Virochip) and deep sequencing, for the identification and characterization of 2009 pandemic H1N1 influenza A virus. Using nasopharyngeal swabs collected during the earliest stages of the pandemic in Mexico, Canada, and the United States (n = 17), the Virochip was able to detect a novel virus most closely related to swine influenza viruses without *a priori* information. Deep sequencing yielded reads corresponding to 2009 H1N1 influenza in each sample (percentage of aligned sequences corresponding to 2009 H1N1 ranging from 0.0011% to 10.9%), with up to 97% coverage of the influenza genome in one sample. Detection of 2009 H1N1 by deep sequencing was possible even at titers near the limits of detection for specific RT-PCR, and the percentage of sequence reads was linearly correlated with virus titer. Deep sequencing also provided insights into the upper respiratory microbiota and host gene expression in response to 2009 H1N1 infection. An unbiased analysis combining sequence data from all 17 outbreak samples revealed that 90% of the 2009 H1N1 genome could be assembled *de novo* without the use of any reference sequence, including assembly of several near full-length genomic segments. These results indicate that a streamlined metagenomics detection strategy can potentially replace the multiple conventional diagnostic tests required to investigate an outbreak of a novel pathogen, and provide a blueprint for comprehensive diagnosis of unexplained acute illnesses or outbreaks in clinical and public health settings.

## Introduction

The 2009 H1N1 influenza A virus (2009 H1N1) is a novel reassortant virus comprised of genomic segments originating from swine, avian, and human influenza strains [1], [2]. After the initial identification of a swine-origin H1N1 by the CDC in April 2009, the novel virus rapidly achieved sustained human-to-human transmission worldwide, prompting a WHO declaration of a level 6 pandemic and rapid development of a vaccine [3]. As of August 6th, 2010, there have been at least 50 million cases and 18,449 confirmed deaths worldwide from the 2009 H1N1 pandemic (http://www.who.int/csr/don/2010_08_06/en/index.html).

The emergence of 2009 H1N1 was marked by diagnostic difficulties that hampered early efforts at detection. Rapid point-of-care diagnostics were insensitive for diagnosing the novel virus, and while existing molecular methods such as RT-PCR were able to detect influenza, they could not differentiate 2009 H1N1 from seasonal H3N2 or H1N1 infection [4]. Initial efforts to characterize the virus relied on traditional Sanger sequencing and the use of PCR primers to highly conserved regions in the influenza genome, methods that would likely have failed had the virus been more genetically divergent [1], [2]. No clinical or laboratory test was initially available to identify 2009 H1N1 with high sensitivity and specificity, and the virus may have circulated undetected in the human population for months [5]. Thus, there is a clear need for the introduction of broad-range viral detection methodologies into clinical and public health settings to deal with emerging threats such as 2009 H1N1 influenza.

Metagenomic approaches, including microarrays and deep sequencing, have proven increasingly successful in recent years for the diagnosis of infectious diseases by novel viral pathogens [6]. The Virochip is a pan-viral microarray that is designed to simultaneously detect all known viruses [7], [8]. Novel viral species or strains can also be detected on the basis of conserved sequence homology to known viruses, as demonstrated by the discovery of the SARS coronavirus by Virochip in 2003 [9], [10]. Other novel viruses previously identified by Virochip include a new clade of rhinoviruses [11], a human cardiovirus associated with respiratory and diarrheal illness [12], and avian bornavirus, the etiologic agent of outbreaks of proventricular dilation disease (PDD) in birds [13], [14]. For detection of known respiratory viruses, the Virochip microarray was found to have a sensitivity and specificity comparable to or superior to conventional diagnostic testing [11], [15]. Deep sequencing is a complementary approach capable of detecting viruses that are too divergent from known viruses to be detected by either PCR or microarray techniques [16], [17], [18], [19], [20], [21], [22]; reviewed in [6]. *De novo* pyrosequencing has enabled the discovery of two novel arenaviruses, an arenavirus associated with a fatal cluster of cases in solid-organ transplant patients (Dandenong virus) [22], and a hemorrhagic fever-associated arenavirus from South Africa (Lujo virus) [21].

Metagenomic approaches are especially attractive in the study of influenza given the constant threat of antigenic drift and shift [23]. Microarrays can rapidly type the origins of different segments to guard against the threat of novel reassortants and to detect co-infections with other pathogens [8], [15], [24], [25], [26], [27], [28], while pyrosequencing or the use of resequencing microarrays can monitor the emergence of mutations that confer virulence or resistance to antiviral drugs [29], [30], [31]. Deep sequencing strategies have been recently employed to detect seasonal influenza viruses in three clinical samples [32], as well as identify quasispecies of 2009 H1N1 in a single autopsy lung sample [33].

Here we analyzed a group of 17 early cases from the 2009 H1N1 pandemic from the United States (California), Canada, and Mexico by Virochip and deep sequencing. We show that the Virochip, in the absence of *a priori* information, was capable of rapid characterization of 2009 H1N1 as an influenza A (H1N1) virus most closely related to swine influenza viruses. We also demonstrate the utility of deep sequencing of clinical samples to identify and characterize not only the novel pathogen but also the microbiota and host response to infection.

## Results

### Clinical data

**Figure 1:**
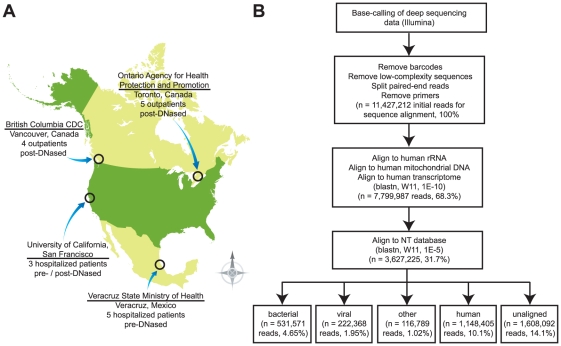
2009 H1N1 sample collection, processing, and analysis. (**A**) Map of North America displaying originating locations for the 17 samples from laboratory-confirmed or suspected 2009 H1N1 cases analyzed in this study. The method for DNase treatment, found to greatly impact the deep sequencing results, is also shown. (**B**) Pipeline for metagenomic analysis of deep sequencing data. Filtered high-quality reads are classified by successive alignments to publicly available sequence databases.

Nasopharyngeal swab samples collected from 17 patients in North America from April to June of 2009 were included in this study (Table S1; Fig. 1A). Study patients were children or young adults, 59% (10 of 17) male, with an average age of 22 years (range 1 to 41 years). Although the majority of cases (65%, 11 of 17) were upper respiratory infections in the outpatient setting, 35% (7 of 17) of patients were hospitalized for presumed H1N1 infection. All three study patients from California were hospitalized in the intensive care unit (ICU) with H1N1-associated pneumonia. Two of the three California patients were pregnant women, a group previously reported at high risk for severe complications from H1N1 infection [34].

### Virochip identification of 2009 H1N1

**Figure 2:**
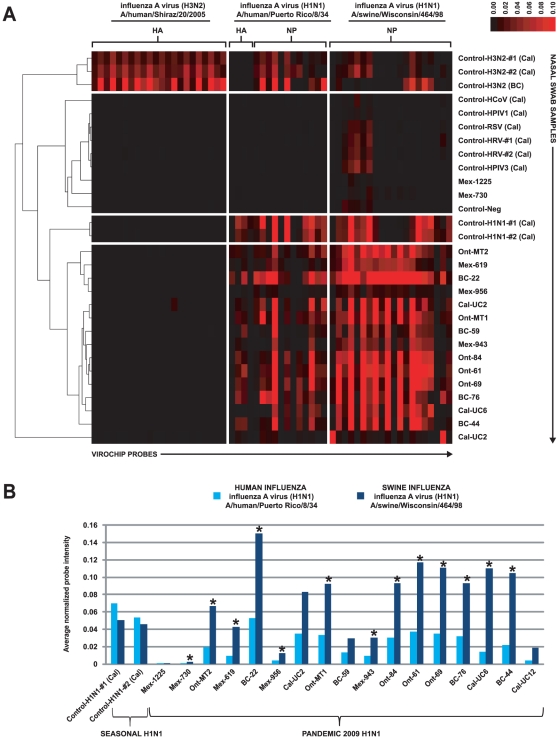
Virochip microarray analysis of samples from patients with influenza-like illness. (A) Cluster analysis and heat map profile for 29 nasopharyngeal swab samples analyzed using the Virochip. Samples (y-axis) and probes on the Virochip designed *a priori* and derived from 3 influenza A strains (x-axis) are clustered using hierarchical clustering. The red color saturation indicates the magnitude of normalized probe intensity. (**B**) For each sample, the average normalized probe intensity for Virochip probes corresponding to human H1N1 influenza A (light blue) or swine H1N1 influenza A (dark blue) is shown. The asterisks denote samples for which the difference in probe intensity is significant by t-test analysis (p<0.05). Abbreviations: HA, hemagglutinin; NP, nucleoprotein; Cal, California; HRV, human rhinovirus; HPIV1, human parainfluenza virus 1; HCoV, human coronavirus; HPIV3, human parainfluenza virus 3; RSV, respiratory syncytial virus; Neg, negative.

To determine whether a pan-viral microarray assay was capable of identifying novel 2009 H1N1 in the absence of *a priori* sequence information, we used the Virochip to comprehensively screen for viruses in 29 nasopharyngeal swab samples from individuals with influenza-like illness. Seventeen samples from Mexico (n = 5), Canada (n = 9), and the United States (California) (n = 3) were suspected or PCR-confirmed cases of H1N1 infection. The remaining control samples, all from California, were positive for H1N1 seasonal influenza (n = 2), H3N2 seasonal influenza (n = 3), another respiratory virus (n = 6), or negative by all diagnostic testing (n = 1). Of note, the probes on the Virochip were designed prior to the emergence of pandemic 2009 H1N1. Virochip hybridization patterns corresponding to 15 of 17 samples suspected or confirmed positive for 2009 H1N1 grouped together by hierarchical cluster analysis, and were clearly distinguishable from patterns corresponding to seasonal H3N2 influenza virus (Fig. 2A). Two of the 17 samples, Mex-1225 and Mex-730, exhibited very weak microarray probe intensities on Virochip analysis and were grouped with the negative control samples. The finding of weak Virochip intensities for these two samples was reproducible, and likely secondary to low viral titers and/or high background from host nucleic acid which can interfere with the random PCR amplification step of Virochip analysis [8], [15]. The average normalized microarray intensities for the 2009 H1N1 samples were more pronounced with H1N1 probes derived from swine influenza (A/swine/Wisconsin/464/98) than with probes derived from human influenza (A/human/Puerto Rico/8/34); the difference was statistically significant for 13 of 17 samples by t-test analysis (Fig. 2B). Microarrays corresponding to seasonal H1N1 influenza displayed the opposite pattern, with H1N1 probes derived from human influenza stronger in intensity than those derived from swine influenza. Minimal cross-hybridization in influenza A probes was observed with samples either negative or harboring other respiratory viruses. The Virochip microarray was also analyzed using Z-score analysis and E-Predict, an automated method for viral species identification in microarrays [7], [35]. Both methods incorporate information from all of the oligonucleotide probes on the Virochip simultaneously, including probes to the full range of influenza strains, and revealed that a virus most similar to swine influenza A (A/swine/Wisconsin/464/98) was present in these samples (data not shown). Thus, the Virochip detected the presence of a virus most closely related to swine influenza A/H1N1 in samples from individuals infected with 2009 H1N1.

### Metagenomics of 2009 H1N1 samples

To further characterize the metagenomics of 2009 H1N1 infection in humans, we labeled the 17 influenza samples positive for 2009 H1N1 by Virochip with distinct molecular barcodes and analyzed them by paired-end deep sequencing on three lanes of an Illumina Genome Analyzer IIx. After trimming reads to remove barcodes and exclude low-complexity or primer sequences, 11,427,212 high-quality 60-bp sequence reads were subjected to an iterative BLASTN analysis pipeline (Fig. 1B). From the initial set of reads, a total of 9,819,120 (85.9%) reads were alignable (word size  = 11, E-value  = 1×10^−10^ or 1×10^−5^) to sequences obtained from the NCBI non-redundant nucleotide (NT) database (March 2010 build).

**Figure 3:**
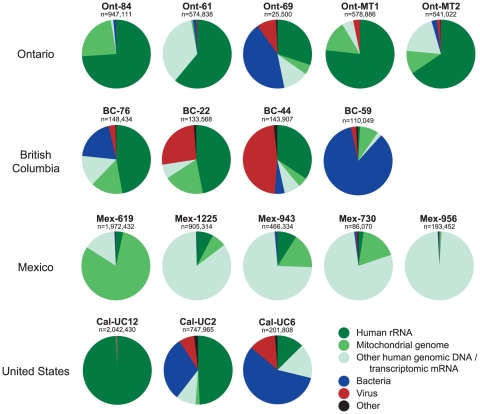
Metagenomic analysis of 2009 H1N1 samples by deep sequencing. Pie charts depicting the number and distribution of sequence reads for each sample. Deep sequencing reads were successively aligned to human rRNA (dark green), human mitochondrial genomic DNA (medium green), other human genomic DNA/transcriptomic RNA (light green), and the non-redundant nucleotide (NT) database, containing bacterial (blue), viral (red), and “other” (black) sequences. Reads designated as “other” consisted solely of artificial plasmids or environmental sequences.

The distribution of reads aligning to human, bacterial, and viral sequences varied greatly depending upon the laboratory preparation method for the clinical sample prior to sequencing (Figure 3). Samples from Mexico were not treated with DNase post-extraction, resulting in most reads from those samples (75 to 98%) aligning to human genomic DNA. In contrast, samples that were treated with DNase post-extraction (United States and Canada) contained many reads aligning to abundant, non-coding RNAs, including human and bacterial ribosomal RNA (rRNA). Despite DNase treatment, reads aligning to the human mitochondrial genome remained abundant for several samples. The overall percentages of bacterial (4.65%), viral (1.95%), other (1.02%) and nonaligning (14.1%) reads were low, with some notable individual exceptions. Approximately 85% of reads from sample BC-59 aligned to bacterial sequences, mostly corresponding to the *Streptococcus* genus. Two samples from British Columbia had a disproportionately large number of reads that aligned to viruses due to the addition of MS2 phage as an internal positive control for the Luminex Respiratory Virus Panel (RVP) assay (Luminex, Austin, TX).

### Deep sequencing of bacteria

**Figure 4:**
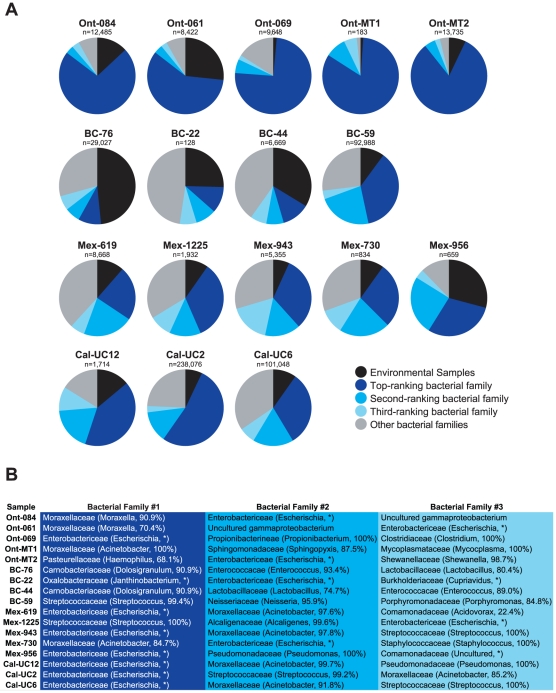
Deep sequencing analysis of bacteria in 2009 H1N1 samples. All bacterial reads aligned to bacterial rRNA and are classified only at the genus level (family level for *Enterobactericeae*). (**A**) The pie charts depict the proportion of bacterial rRNA reads corresponding to the top-ranking (dark blue), second-ranking (medium blue), and third-ranking (light blue) bacterial families, as well as other remaining families (grey) and unidentified environmental samples (black). (**B**) For each sample, the identities of the top-ranking, second-ranking, and third-ranking bacterial family, as well as the majority genus within each family, are shown.

To determine if alterations in the upper respiratory tract microbiome were present in samples from patients infected with H1N1 virus, we further analyzed the deep sequencing reads aligning to bacteria. Interestingly, all bacterial reads aligned to rRNA sequences, likely due to the high relative abundance of rRNA transcripts in bacteria [36], and no bacterial mRNA reads were observed. Given the high degree of conservation of rRNA sequences in bacteria, each read could only be unambiguously classified at the genus level (or at the family level in the case of *Enterobactericeae*) (Fig. 4). In general, a high level of diversity of bacterial families was observed in different samples, reflecting the known diversity of bacterial flora in the nasopharynx [37]. Reads to the top three bacterial families comprised anywhere from 22 to 97% of all bacterial reads, depending on the sample (Fig. 4A). *Moraxellaceae* or *Enterobactericeae* were the most common bacterial families found, accounting for one of the three most common bacterial families in 14 of 17 samples (Fig. 4B). Significant numbers of reads aligning to the *Streptococcus* genus were only seen in 5 of 17 samples, while reads to unculturable agents (“environmental samples”) accounted for between 1 to 48% of sequences aligning to bacteria.

### Deep sequencing of 2009 H1N1 influenza and other viruses

The majority of viral reads (n = 122,487, 55.1%) aligned to members of the *Orthomyxoviridae* (influenza) family (Table 1). Phages comprised the second most common group of viruses, including known controls (e.g. MS2 control phages), trace reagent contaminants (e.g. bacteriophage M13), and environmental phages. Reads aligning to plant viruses were observed in two samples (BC-76 and Mex-1225). Sixteen reads to murine leukemia viruses (MLVs) were detected in 4 of 17 samples. In general, these MLV reads aligned slightly better to Moloney murine leukemia virus (MMLV) than to the recently described human xenotropic murine leukemia-related virus (XMRV) [38], [39], [40], although, for some reads, the alignments were identical. Interestingly, a single overlapping 97-nt paired-end read from BC-22 aligned with 98% identity to a filovirus, Ebola-Sudan (97% query coverage, E-value  = 9×10^−39^) and mapped to a gene coding for the Ebola viral glycoprotein. This BC-22 read also aligned to other filoviruses (69–98% identity) but did not align to any other viral family. We were able to reproduce detection of this single filovirus read by specific RT-PCR, but, despite the use of multiple sets of primers, we were unable to recover additional filovirus sequence from this sample (data not shown). Aside from influenza, the only other respiratory viral pathogen detected was human adenovirus 4 in one Canadian sample (BC-22, 26 reads).

**Table 1:** Distribution of viral reads in 2009 H1N1 influenza samples.

SAMPLE	TOTAL ALIGNED READS	TOTAL VIRAL READS	FLU	% FLU (TOTAL ALIGNED READS)	% FLU (VIRAL READS)	PHAGE	MLV	OTHER VIRAL
**Ont-61**	574,838	2,649	2,649	0.46%	100%	-	-	-
**Ont-69**	25,500	2,243	2,243	8.8%	100%	-	-	-
**Ont-84**	947,111	884	884	0.091%	100%	-	-	-
**Ont-MT1**	578,886	18,079	18,079	3.1%	100%	-	-	-
**Ont-MT2**	541,022	8,594	8,594	1.6%	100%	-	-	-
**BC-22**	133,568	35,374	6,036	4.5%	17.1%	29,337	-	1 baculovirus
**BC-44**	143,907	67,448	1,824	1.3%	2.7%	65,624	-	-
**BC-59**	110,049	2,751	18	0.016%	0.65%	2,730	-	3 HERV
**BC-76**	148,434	4,871	4,835	3.3%	99.3%	6	-	26 adenovirus type 4; 2 ebola virus; 2 sweet potato chlorotic stunt virus
**Mex-619**	1,972,432	1,050	938	0.048%	89.3%	111	1	-
**Mex-730**	86,070	81	78	0.091%	96.3%	3	-	-
**Mex-943**	466,334	433	415	0.089%	95.8%	16	2	-
**Mex-956**	193,452	19	6	0.0031%	31.6%	5	-	8 HERV
**Mex-1225**	905,314	21	10	0.0011%	47.6%	10	-	1 iridovirus (rice virus)
**Cal-UC2**	747,965	54,081	53,671	7.2%	99.2%	393	5	12 baculovirus
**Cal-UC6**	201,808	23,516	21,937	10.9%	93.3%	1,571	8	-
**Cal-UC12**	2,042,430	274	270	0.013%	98.5%	4	-	-
**TOTAL**	9,819,120	222,368	122,487	1.2%	55.1%	99,810	16	55

Abbreviations: Orthomyxo, orthomyxovirus; MLV, murine leukemia virus; HERV, human endogenous retrovirus.

**Figure 5:**
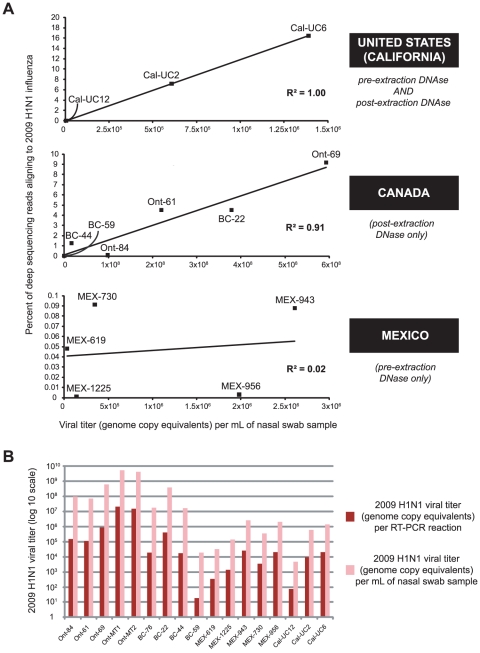
Relationship between percentage of deep sequencing reads aligning to 2009 H1N1 and viral titer. (**A**) For each sample, the percentage of deep sequencing reads aligning to 2009 H1N1 is plotted as a function of the calculated viral titer (genome copy equivalents) per mL of nasal swab sample, and a linear regression line is fitted to the data. Samples are stratified by originating location and method of DNase treatment. (**B**) For each of the 17 samples, the calculated 2009 H1N1 viral titer (genome copy equivalents) per RT-PCR reaction (dark red) or per mL of nasal swab sample (pink) is shown.

Influenza reads aligning to *Orthomyxoviridae* were found in all specimens, ranging from 6 to 53,671 reads, or 0.00011% to 10.9% of all aligning reads. After stratifying by originating location and corresponding method of sample processing (pre-DNase and/or post-DNase treatment), the percentage of total reads aligning to influenza was linearly correlated with calculated viral titers by real-time quantitative RT-PCR for sites in the United States (California) and Canada but not in Mexico (Fig. 5A). Treatment with DNase twice yielded the best percentage recovery of influenza viral sequences (Fig. 5A, “United States (California)”), though only three samples were treated as such. The two samples with the lowest viral titers (quantified based on RNA genome copy equivalents) were Cal-UC12 and BC-59, with 71 and 17 copies per RT-PCR reaction corresponding to 1.82×10^4^ and 4.74×10^3^ copies per mL of nasal swab sample, respectively (Fig. 5B). However, despite very low viral titers, H1N1 influenza was still detectable by deep sequencing, with 270 and 18 reads aligning to influenza for Cal-UC12 and BC-59, respectively (Table 1).

**Figure 6:**
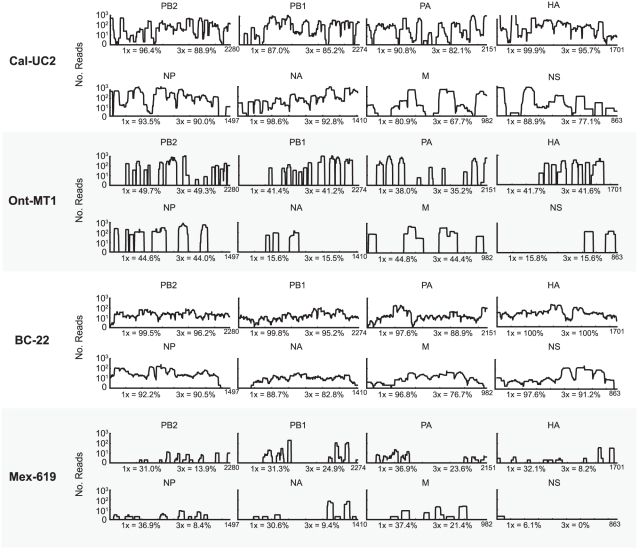
Coverage map of the influenza genome for four 2009 H1N1 samples. For each originating location, the sample that was found to have the best coverage is shown. The coverage is plotted on a log scale as a function of nucleotide position. For each segment, the coverage percentages at 1X and 3X are indicated.

Influenza reads from all 8 segments were found in 7 of 17 samples, while reads from at least two different segments were found in every sample (Fig. 6; Figs. S1 and S2). Interestingly, reads to the NS (nonstructural) segment were significantly less likely to be found than reads to other segments (p<0.001 by Chi-square analysis), with NS reads detected in only 8 of 17 samples. In contrast, reads to PB2 were found in all 17 samples. Samples from Mexico had the worst overall coverage, likely due to the high percentage of human genomic background sequences as a result of not treating samples with DNase post-extraction. In the sample with the best overall coverage (BC-22), 97.0% of the 2009 H1N1 genome had at least 1x coverage, while 91.5% of the genome had at least 3x coverage.

### Sanger sequencing of 2009 H1N1 and analysis of single nucleotide polymorphisms (SNPs)

We sought to assess the accuracy of deep sequencing relative to traditional Sanger sequencing as well as search for single nucleotide polymorphisms (SNPs) in the assembled deep sequencing reads that may represent heterogeneous populations of influenza. Deep sequencing reads from the two samples in the study with the highest coverage of the 2009 H1N1 genome, BC-22 (97.1% coverage at 1X) and Cal-UC2 (92.6% coverage at 1X), were mapped to their full-genome scaffolds obtained by Sanger sequencing. Overall, there was a high degree of concordance (>99.9%) with deep sequencing and traditional Sanger sequencing for both BC-22 and Cal-UC2 (Table S2).

Next, to identify potential mutations at key sites in the genome that mediate drug resistance and virulence, we compared the genomes of BC-22 and Cal-UC2 with the full-genome reference sequence A/California/06/2009(H1N1) (Table S2). In total, there were 30 nonambiguous nucleotide differences between BC-22 and the original California strain and 39 nonambiguous nucleotide differences between Cal-UC2 and the original strain, of which 80% and 85% were detected by Sanger sequencing and 77% and 72% by deep sequencing alone, respectively. These nucleotide differences corresponded to 12 and 18 nonsynonymous mutations in the BC-22 and Cal-UC2 genomes, respectively. The genome segment with the most amino acid changes was the HA gene (27 of 69 nucleotide differences, or 39.1%). For both BC-22 and Cal-UC2, there were no differences from the reference sequence A/California/06/2009(H1N1) at key sites in the genome corresponding to known mutations for antiviral resistance, enhanced transmission, or increased virulence.

### Analysis of transcriptome reads in 2009 H1N1 samples

**Figure 7:**
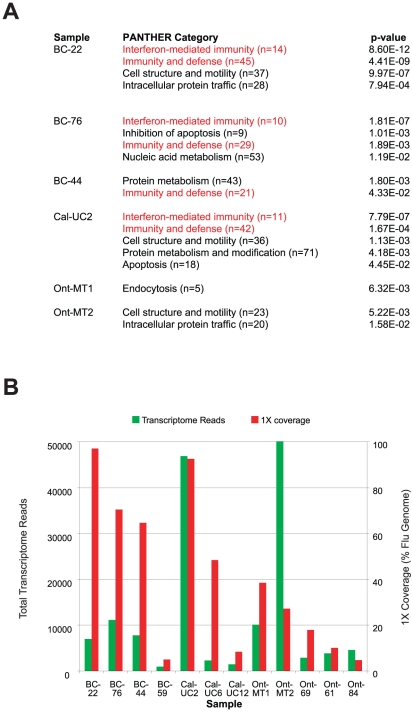
Deep sequencing analysis of the human transcriptome in 2009 H1N1 samples. Transcriptomic analysis was performed on the 12 samples that were treated with DNAse post-extraction. (**A**) Overexpressed genes in 2009 H1N1 samples relative to control samples were categorized using the PANTHER database [52]. The six samples (out of 12) containing categories that are significantly overrepresented (p<0.05) are displayed. Categories related to immunity and host defense are highlighted in red. (**B**) Bar graph showing the total reads aligning to the transcriptome (green) and percentage coverage of the 2009 H1N1 genome by deep sequencing (red).

Although samples were prepared principally for the purposes of metagenomics and the detection of known or novel pathogens, treatment with DNase post-extraction to discriminate host mRNA from genomic DNA enabled analysis of the transcriptomics of 2009 H1N1 infection in 12 of the 17 nasopharyngeal swab samples. Deep sequencing reads aligning to the RefSeq RNA database in these 12 samples were considered to arise from mRNA transcripts. Since no RNA-seq data corresponding to nasopharyngeal samples from healthy individuals can be found in the NCBI Sequence Read Archive (http://www.ncbi.nlm.inh.gov/sra), we used a set of nasopharyngeal swab samples collected from 11 Kleine-Levin Syndrome (KLS) patients with influenza-like illness but testing negative for influenza as background controls. Genes associated with immunity and interferon were significantly up-regulated (p<0.05) in four of the 12 influenza samples as compared to the control samples, and from two study sites with different specimen processing protocols (Fig. 7A). The interferon-related genes that were preferentially up-regulated in 2009 H1N1 samples relative to controls coded for chemokine ligands, 2′-5′ oligoadenylated synthetases, IFN-inducible proteins, and STAT proteins, proteins previously shown to play essential roles in the interferon-mediated host immune response to viral infection [41]. Up-regulation of interferon and immunity genes was seen in the four samples with the highest 1X coverage of the influenza genome but not necessarily in samples with a high number of total transcriptome reads (Fig. 7B). Genes associated with cell structure and motility as well as protein metabolism and intracellular protein trafficking were also upregulated in 2009 H1N1 samples relative to control samples (Fig. 7A).

### *De novo* assembly of 2009 H1N1

**Figure 8:**
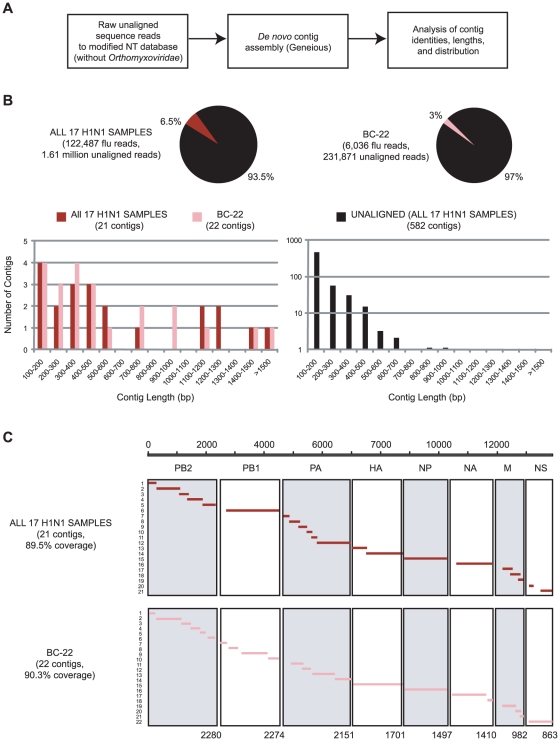
*De novo* assembly of the 2009 H1N1 genome. (**A**) Algorithm for *de novo* assembly of the 2009 H1N1 genome assuming no *a priori* knowledge of the *Orthomyxoviridae* family and thus, no available reference genome. (**B**) Plot of number of contigs vs. contig length for *de novo* assembled contigs mapping to 2009 H1N1 influenza derived from all 17 pooled samples (dark red) or sample BC-1422 alone (pink), as well as for unaligned contigs (black). Note that the longest contigs all map to 2009 H1N1, even though influenza reads in all 17 pooled samples or BC-1422 comprise only 7.6% or 2.6% of the total reads, respectively. (**C**) Coverage of the 2009 H1N1 genome by *de novo* contig assembly from reads corresponding to all 17 pooled samples (top, dark red lines) or BC-1422 alone (bottom, pink lines).

We hypothesized that the unprecedented depth of coverage obtainable by deep sequencing could enable detection and even *de novo* assembly of novel viral pathogens in the absence of any reference genome. To demonstrate the feasibility of this approach in elucidating the cause of outbreaks from hitherto novel or unknown viruses, we generated a modified NT database from which all sequences corresponding to the *Orthomyxoviridae* family had been removed (Fig. 8A). Pure *de novo* assemblies of paired-end reads pooled from all 17 2009 H1N1 samples which did not align to the modified NT database were then constructed using Geneious software (Geneious, Auckland, NZ). Of the 582 final contigs (contiguous sequences) of length greater than 100 bp generated from the *de novo* assembly, 21 contigs mapped onto a reference sequence of 2009 H1N1 influenza, with an N50 contig size of 1,167 bp and coverage of 89.5% of the genome (Fig. 8B). The four longest contigs all corresponded to 2009 H1N1 and included the nearly full-length sequences of the PB1 (polymerase), NP (nucleoprotein) and NA (neuraminidase) segments (Fig. 8C). A cursory analysis of the remaining 561 contigs that did not map to the influenza genome revealed that ∼90% of these sequences were low-complexity sequencing artifacts. From a similar analysis performed on the best single sample (BC-22), *de novo* assembly of 90.3% of the genome was possible. No misassemblies were observed even though the percentage of *Orthomyxoviridae* reads from all 17 H1N1 samples or BC-22 alone comprised only 7.6% or 2.6% of the corresponding unaligned reads to the modified NT database, respectively.

## Discussion

In this study, we demonstrate the utility of a metagenomics-based strategy combining a rapid, broad-spectrum diagnostic assay (the Virochip microarray) with comprehensive deep sequencing to identify and characterize a novel outbreak pathogen, using pandemic 2009 H1N1 influenza as a case example. The Virochip was capable of differentiating seasonal H3N2 and H1N1 influenza from the novel 2009 pandemic strain by the use of multiple probes designed *a priori*, and accurately characterized the novel virus as closely related to swine influenza viruses. Complementary deep sequencing of 17 clinical samples collected early in the course of the 2009 H1N1 pandemic enabled detection of reads to the novel strain in every sample, as well as *de novo* assembly of ∼90% of the genome amidst an enormous background of unaligned sequence reads. As with the SARS coronavirus, the lack of a broad-based diagnostic test that was sufficiently sensitive and specific likely contributed to the delayed identification of 2009 H1N1 as the cause of an initial outbreak of influenza-like illness in Mexico. Our approach – using the Virochip microarray for rapid screening and deep sequencing for *de novo* assembly and more detailed characterization – is robust, comprehensive, and directly applicable for the investigation of future outbreaks from novel viral pathogens, especially those which do not grow in culture.

Nearly all previous studies of metagenomic sequencing of viruses from human clinical material for detection and discovery have employed pyrosequencing [6], [16], [17], [18], [19], [20], [21], [22], [32], [42], [43]. The longer reads from pyrosequencing (250–450 bp) facilitate the assembly of individual reads into contigs, which assists in the classification of reads by homology-based BLAST alignments. Our results suggest, for example, that longer-read sequencing methods such as pyrosequencing may be better suited than Illumina sequencing for identification of bacteria. Accurate discrimination of bacteria at the species (or even genus) level was not possible with short 60-bp Illumina reads (Fig. 4B), as rRNA sequences corresponding to related bacteria were found to be essentially 100% identical over a 60-bp range. We did not *de novo* assemble the short Illumina reads aligning to bacterial rRNA into longer, more easily identifiable contigs given the known presence of multiple related bacterial species in the nasopharynx and the very high (and likely) risk of producing misassemblies. On the other hand, a key advantage of Illumina sequencing is the significant increase in read depth (∼100-fold) relative to pyrosequencing. The greater depth of sequencing provides better and more accurate genome coverage as well as increased capability to multiplex clinical samples.

In our study of 2009 H1N1 influenza by deep sequencing, Illumina read lengths of 60 bp were sufficiently long to accurately classify the vast majority of individual reads, thus making *de novo* contig assembly prior to alignment unnecessary. In addition, we were limited by the computational resources required for *de novo* assembly of ∼11.5 million sequences in a reasonable amount of time. Our results demonstrate that a computational pipeline consisting of sequential alignments of individual reads using high-stringency cutoffs (Fig. 1), followed by *de novo* assembly of the remaining unaligned reads and detailed analysis of the resulting contigs (Fig. 8), is an efficient strategy for analyzing the large datasets generated by Illumina sequencing of clinical samples and for pathogen discovery. In particular, the final *de novo* assembly step is critical in identifying divergent sequences that may correspond to novel pathogens. The assembly of longer contigs also eases the burden of computationally intensive amino acid comparison algorithms such as BLASTX, TBLASTX and PHI-BLAST by reducing the number of sequences that need to be analyzed.

Although the costs of deep sequencing (∼$200-$1000 USD/sample) are still much greater than the reagent costs for diagnostic RT-PCR assays (∼$10 USD), deep sequencing is able to generate much more useful information including data on the microbiome and host gene expression as well as whole-genome sequencing of pathogens which would require, for example, at least $1,200 USD per influenza genome via traditional methods. In addition, unbiased deep sequencing allows broad-based detection of novel viruses that may elude diagnosis by specific RT-PCR assays. With paired-end read lengths achievable on the Illumina platform now approaching 300 bp, the use of deep sequencing for identification of even highly divergent pathogens at exceedingly low titers becomes feasible.

The number of reads corresponding to 2009 H1N1 and the ability to map sequences onto the genome were highly dependent on the method of sample preparation. The fewest viral reads were detected in the five samples from Mexico that were not treated with DNase after nucleic acid extraction. These samples contained the highest percentage of human genomic sequence, with over 90% of sequences aligning to the human genome, as well as the fewest reads to bacteria. Nakamura et al. also found >90% host genomic material in nasopharyngeal aspirates from influenza-infected individuals, and detected a similarly low percentage of reads to influenza, despite the use of centrifugation to remove bacterial and cellular material [32]. Our data indicate that one of the most important steps in viral detection by deep sequencing may be the removal of host genomic DNA by post-extraction treatment with DNase – a relatively straightforward procedure for most clinical laboratories.

As post-extraction DNAse treatment was not 100% efficient (with the observation of residual DNA mitochondrial reads in several samples), it is true that additional pre-processing by filtration, ultracentrifugation, or density-gradient purification would likely have resulted in even better enrichment of viral sequences [6]. However, for this metagenomics study, we sought to mimic as closely as possible a clinical/public health laboratory setting, and such enrichment strategies may be labor-intensive and neither conducive to the demands of clinical laboratories nor practical given the limited amount of clinical sample available. In addition, an important drawback of directed viral purification methods is that by biasing the sample towards virus detection, they can severely hinder the ability to detect potential non-viral pathogens or investigate patterns of human gene expression in response to viral infection, as we have done here.

It has been suggested that co-infections with *Streptococcus pneumoniae* may impact the severity of the clinical presentation in patients infected with 2009 H1N1 [44]. Remarkably, very few co-pathogens, either viral or bacterial, were found among the 17 samples chosen for analysis. Deep sequencing revealed that only five samples had a significant number of bacterial reads to the *Streptococcus* genus, which may either represent potential pathogens (e.g. *Streptococcus pneumoniae*), or simply normal human nasopharyngeal viral flora (e.g. *Streptococcus oralis, sanguis*, and/or *mitis*). One sample from a Canadian patient with an upper respiratory infection from 2009 H1N1 (BC-22) did contain a small number of reads from adenovirus type 4 (n = 26). These results imply that co-pathogens did not play a significant role in infection by 2009 H1N1 influenza in the 17 samples analyzed. Further study of a much larger sample set will be needed to fully ascertain the role of co-pathogens in 2009 H1N1 infection.

Although 4 of 17 samples contained reads to murine leukemia viruses, it appears more probable on the basis of sequence alignments that the source of these reads is trace MMLV vector contamination of the reverse transcriptase enzyme used in preparing libraries for deep sequencing rather than infection by XMRV, a gammaretrovirus associated with prostate cancer and chronic fatigue syndrome in humans [39], [40] and recently detected in the respiratory tract [38]. In addition, one sample (BC-22) contained a paired-end read of 97 nt that was a 98% match to the filovirus Ebola-Sudan. We considered this most likely a laboratory contaminant, since we did not identify any other reads to filoviruses and were unable to amplify additional filovirus sequence from BC-22. However, the origin of this sequence ultimately remains unknown, as we could not subsequently detect it in any sample or reagent by specific RT-PCR, and none of the laboratories involved in the analysis works with Ebola virus. We also found reads corresponding to plant viruses in two samples (BC-76 and Mex-1225). Detection of plant viruses in nasal swab samples is not unexpected given that the nasopharynx is contiguous with the oropharynx, and that an abundance of plant viruses of presumed dietary origin can be found in the human digestive tract [45]. Our discovery of unexpected viral reads corresponding to murine leukemia viruses, filoviruses, and plant viruses in the deep sequencing data underscores the challenges of analyzing rare or unique sequences that may correspond to colonizing flora, reagent/sample contamination, or even true pathogens.

Using deep sequencing, we were able to recover and assemble near full-length genome sequences of the 2009 H1N1 virus from two individual patient specimens. Since the molecular determinants of influenza pathogenesis have been well-studied, we were able to analyze the genomic sequence for single nucleotide polymorphisms (SNPs) that specifically correlate with antiviral resistance, enhanced transmission or increased virulence. For a novel virus, where such genotypic findings would not yet be correlated with phenotypic data, the overall sequence and structure of the genome may still yield valuable insights into viral transmission and mechanisms of pathogenesis.

Analysis of transcriptome reads demonstrated that immunity and interferon genes were significantly up-regulated in 4 nasopharyngeal swab samples from 2009 H1N1-infected patients as compared to background controls. Interestingly, these 4 samples did not necessarily have a high number of transcriptome reads, but did have the highest 1X coverage of the influenza genome (Fig. 7B). Coverage of the influenza genome may be the best proxy measure for influenza activity and the associated host response in deep sequencing analysis, as it is relatively independent of PCR duplication artifacts that can be introduced during sample preparation for deep sequencing [46]. Our finding of increased expression of interferon-associated host genes in clinical samples from patients with 2009 H1N1 infection is intriguing and consistent with very recent data showing that the NS1 protein of 2009 H1N1 lacks the ability to block general host gene expression in both human and swine cells *in vitro* [47]. Further studies are needed to assess the clinical relevance of increased interferon-associated host gene expression in 2009 H1N1 infection *in vivo*. Nevertheless, this finding highlights the power of an unbiased deep sequencing approach by not only facilitating detection of the viral pathogen but also enabling elements of the host response to be simultaneously interrogated, thus generating new hypotheses for analysis of host-pathogen interactions.

Our study is the first to explore the relationship between viral titer and depth of sequencing required to detect a candidate viral pathogen. In samples that are treated with DNase after nucleic acid extraction (“post-DNAse”), there is a linear correlation between percentage of viral reads in the deep sequencing data and viral titers as estimated by quantitative RT-PCR (Fig. 5A). There is no such observed correlation if samples do not undergo post-DNAse treatment, likely secondary to high residual host genomic background, and thus these findings may only apply to RNA viral targets. However, it is encouraging that sequence reads corresponding to 2009 H1N1 were detectable in all 17 samples, even in those samples with very low viral titers approaching the analytical limits of detection for RT-PCR assays (i.e. Cal-UC12 and BC-59) (Fig. 5B).

More studies are clearly needed on the metagenomics of different viral agents (e.g. RNA vs. DNA viruses; lysogenic vs. lytic, etc.) across different stages of infection (e.g. pre-symptomatic, acute, or chronic) and in different types of clinical samples (e.g. respiratory secretions, stool, blood, cerebrospinal fluid, tissue etc.). In addition, although the Virochip microarray has a 12 to 24 hour turnaround time and can be rapidly deployed as a comprehensive screen for viral pathogens in the setting of outbreaks, the complementary deep sequencing approach is currently limited by the time necessary to perform a full paired-end sequencing run on an Illumina Genetic Analyzer IIx (∼1 week) and to analyze the data on a highly parallel computational platform running 32 cores simultaneously (∼1 week). Nevertheless, the data that can be obtained in days to weeks by microarrays and deep sequencing would take months to years using conventional methods. Furthermore, third-generation sequencing technologies which can generate deep sequencing data within hours are now available [48], [49]. The results described here provide a blueprint for the eventual use of microarrays and sequencing as routine diagnostic tools for pathogens in clinical and public health laboratories.

## Methods

### Ethics statement

Cases of influenza-like illness in Canada and Mexico that were confirmed or suspected positive for 2009 H1N1 infection were identified by each individual state public health agency. Informed consent was not obtained as the analysis of samples for pathogens is part of the mandate of clinical testing for each individual agency, and samples as well as demographic, clinical, and laboratory data were de-identified prior to analysis. Cases in California were enrolled in a viral diagnostics study approved by the University of California, San Francisco (UCSF) institutional review board (IRB) (Protocol #H9187-32565), and written informed consent was obtained from all study participants and/or their legal guardians. For all cases, collected samples were analyzed under protocols approved by the UCSF IRB (Protocol #H49187-32368).

### Sample identification and collection

Cases of influenza-like illness in Canada and Mexico were reported by providers and hospitals to the British Columbia Centre for Disease Control (BC-CDC), the University of Toronto/Ontario Agency for Health Protection and Promotion (OAHPP), and the Veracruz Ministry of Health. Suspected or confirmed H1N1 cases were identified through routine laboratory surveillance under protocols approved by each individual state public health agency. For each case, non-identifying demographic and clinical data were reported on standardized forms. Cases in California were identified by infectious disease physicians at University of California at San Francisco (UCSF), and demographic/clinical data were abstracted from the medical record.

### Sample analysis

Nasopharyngeal swab samples collected in viral transport media from 17 patients with either laboratory-confirmed or suspected cases of 2009 H1N1 influenza virus were analyzed. Twelve additional nasopharyngeal swab samples from California were included as negative controls for the Virochip. RNA extractions were performed differently using established protocols specific to individual laboratories. For Mexico, 200 µL of sample with linear polyacrylamide (Ambion, Inc., Austin, TX) added as an RNA carrier was treated with Turbo DNase (Ambion, Inc., Austin, TX)) for 30 minutes at 37°C (“pre-DNase” treatment); nucleic acid was then extracted using the PureLink RNA-DNA kit (Invitrogen, Carlsbad, CA) according to the manufacturer's instructions. For British Columbia and Ontario, 200 µL and 250 µL, respectively, of sample were extracted using the NucliSENS easyMag automated extraction system and then treated with Turbo DNase (“post-DNase” treatment). For California, 200 µL of sample was treated with Turbo DNase, nucleic acid was extracted with the Zymo Viral RNA kit (Zymo Research, Orange, CA), and then the extracted nucleic acid was treated again with Turbo DNase (“pre-/post-DNase” treatment).

### Microarray screening

Total RNA was reverse-transcribed to cDNA, amplified by a modified Round A/B random PCR method, and labeled with Cy3 or Cy5 fluorescent dye as previously described [7], [8]. The labeled samples were normalized to 10 pmol of incorporated dye and hybridized overnight using the Agilent Gene Expression Hybridization kit according to the manufacturer's protocol (Agilent Technologies, Santa Clara, California). The microarray slides used were custom-designed 8×15k or 8×60k arrays synthesized by Agilent Technologies, each containing 11,956 Virochip 70-mer oligonucleotide probes in common. These 11,956 common probes include conserved as well as specific probes representing all viral species in GenBank, and were derived from a previous 2008 Virochip design [12]. The Virochip is not yet commercially available, but is currently implemented as a core diagnostic service of the UCSF Viral Diagnostics and Discovery Center (http://vddc.ucsf.edu). Slides were scanned at 2 µm resolution in XDR (extended dynamic range) mode using an Agilent DNA Microarray Scanner. Virochip microarrays were analyzed with hierarchical cluster analysis, E-Predict, and Z-score single oligonucleotide analysis as previously described [7], [11], [35]. Average normalized microarray intensities were analyzed using a two-sample, two-tailed t-test for unequal variances. All Virochip microarrays used in this study were submitted to the NCBI GEO database (study accession number GSE24034; microarray accession numbers GSM591597-591641; microarray design accession numbers GPL10897 for the 8×15k array and GPL10896 for the 8×60k array).

### PCR and Sanger sequencing

Sequences corresponding to each influenza segment were obtained by one-step RT-PCR using either a Qiagen One-Step RT-PCR kit (Qiagen, Valencia, CA) or an Invitrogen SuperScriptIII One-Step RT-PCR kit with High Fidelity Platinum Taq (Invitrogen, Carlsbad, CA), with primers designed based on conserved terminal and central regions of each segment (Table S3). Reactions run with the Qiagen kit were done in duplicate to account for potential polymerase errors. PCR products were run on a 1.5% agarose gel, and bands of the correct size were cut and extracted. Fragments were cloned into a pCR2.1 vector (Invitrogen, Carlsbad, CA) and Sanger sequenced (Elim Biopharmaceuticals, Hayward, CA) with M13F and M13R primers as well as the original conserved PCR primers. At least three-fold redundancy was obtained for each segment. The whole-genome sequences of 2009 H1N1 samples BC-22 and Cal-UC2, including all 8 segments, have been submitted to GenBank (GenBank accession numbers CY073781-CY073788 for BC-22 and CY073789-CY073796 for Cal-UC2).

### Quantitative RT-PCR

Quantitative RT-PCR for influenza was performed with primers based on conserved regions in the HA segment, H1N1-HA-275F (5′- GGGAAATCCAGAGTGTGAATCACT-3′) and H1N1-HA-375R (GCTCTCTTAGCTCCTCATAATCGATG-3′) using a Stratagene MX3005P Real-Time PCR system. The PCR was performed using a Qiagen One-Step RT-PCR kit (Qiagen, Valencia, CA) in a 12.5 µl reaction containing 3.25 µl H_2_O, 2.5 µl 5X PCR buffer, 2.5 µl Q solution, 0.5 µl dNTP, 0.5 µl RT/Taq enzyme mix, 0.75 µl of each primer, 0.25 µl SYBR Green and 1.5 µl total RNA. Conditions for the PCR were 50°C for 30 min, 95°C for 15 min; 40 cycles of 95°C for 30 s, 50°C for 30 s, and 72°C for 1 min; and a final extension at 72°C for 7 min. The viral titer was estimated using a standard curve derived from an *in vitro*-transcribed and quantified influenza mRNA standard (data not shown). For the Canadian samples, viral titers as determined through Ct values were compared with real-time RT-PCR data obtained independently from reference laboratories in Vancouver (BC-CDC) and Toronto (OAHPP) to check for accuracy (data not shown).

### Deep sequencing library generation

Libraries for deep sequencing were prepared from amplified cDNA libraries using previously published protocols [50]. Briefly, libraries were cleaved with Type IIs restriction endonucleases (*GsuI*), and truncated adapters containing unique 3 or 6 bp molecular barcodes were ligated on the resulting strand ends. Full-length adapters were subsequently added via an additional 15 to 25 cycles of PCR. Libraries were size-selected on a 4% polyacrylamide gel at approximately 350 bp average length, and then loaded at a final concentration of 10 pM on three lanes of a second-generation Genome Analyzer IIx (Illumina, San Diego, CA). Paired-end reads were sequenced for 67 cycles in each direction.

### Deep sequencing analysis

Fluorescent images were analyzed using the Illumina base-calling pipeline 1.5.0 to obtain paired-end sequencing data. Paired-end reads were subsequently classified by strict barcodes, filtered to eliminate low-complexity sequences with an LZW compression ratio cutoff of 0.4, split into individual reads, and stripped of any remaining primer sequences using BLASTN alignments (word size  = 11, E-value  = 1×10^−10^). BLASTN alignments to publicly available sequence databases were performed using four ExtraLarge High CPU-instance Amazon Elastic Cloud Computing (EC2) servers comprising a total of 32 core processors, with an approximate computational time of 24 hours per sequencing lane. Sequence reads that aligned to human rRNA and mitochondrial genome by BLASTN (word size  = 11, E-value  = 1×10^10^) were initially removed. Remaining reads were then classified by successive BLASTN alignments to the RefSeq human transcriptome database (word size  = 11, E-value  = 1×10^−10^, September 2009 build [release 37]) and NCBI non-redundant nucleotide (NT) database (word size  = 11, E-value  = 1×10^−5^, March 2010 build). All reads that aligned to NT were sorted by their best hit into their respective taxa for analysis. Reads that aligned to NT but that did not align to human, bacterial, or viral sequences were categorized as “other”, and consisted solely of environmental sequences or plasmids/artificial constructs. The presence of murine leukemia virus and Ebola virus in the deep sequencing libraries was independently confirmed by specific RT-PCR and direct sequencing (data not shown). We also separately performed directed BLASTN alignments of the original set of unclassified deep sequencing reads to an influenza sequence database (word size  = 11, E-value  = 1×10^−5^) to verify that no influenza reads were incorrectly classified as human or other nonviral sequence (data not shown). All high-quality sequence reads have been submitted to the NCBI Sequence Read Archive (http://www.ncbi.nlm.nih.gov/sra) (accession number SRA023755).

### Transcriptome analysis

Reads corresponding to the human mRNA transcriptome were counted by gene and compared to transcriptome reads from nasopharyngeal swab samples from Kleine-Levin syndrome (KLS) patients with influenza-like illness. KLS is a neuropsychological disorder marked by hypersomnolence, hyperphagia, and hypersexuality usually found in adolescent boys [51]. An ongoing metagenomic analysis of samples from 11 patients with KLS (“KLS samples”) by deep sequencing found only 24 reads to a single rhinovirus A isolate in one sample and no reads to influenza in any sample; in addition, all KLS samples were negative for both pandemic and seasonal influenza by specific RT-PCR (data not shown). As such, it was thought that the KLS samples could serve as appropriate background controls from individuals with influenza-like illness (but testing negative for influenza) for the purposes of transcriptomic analysis. In particular, the KLS samples were derived from a similar demographic as the H1N1 samples (children and young adults) and were processed identically for deep sequencing.

The transcriptomic analysis was performed as follows. First, we selected all genes in RefSeq that were significantly overexpressed in 2009 H1N1 nasopharyngeal swab samples relative to KLS background samples (p<0.05, by Bonferroni-corrected Fisher's Exact Test). To identify overrepresented categories of genes, we inputted the overexpressed RefSeq genes into the PANTHER (Protein Analysis Through Evolutionary Relations) database (http://panther.appliedbiosystems.com) and compared them to a human reference list of transcribed genes [52]. Categories of genes significantly up-regulated in the influenza data relative to the human reference list (p<0.05, by Bonferroni-corrected Fisher's Exact Test) were reported.

### Detection of single nucleotide polymorphisms (SNPs)

SNP analysis of sequence reads mapping to influenza was performed using the SSAHA2 software package [53]. Both paired-end read information and FASTQ-formatted quality scores were incorporated in the calling of SNPs. SNP calling was performed at very high stringency to ensure accuracy. Specifically, the Illumina deep sequencing data had a calculated overall error rate of <1% by analysis of the PhiX control lane (Illumina Inc., Hayward, CA), and at least 5-fold coverage and a quality score of 30 were required for mapping of deep sequencing reads at any given position in the genome. A minority SNP was defined as present if occurring ≥25% of the time at a given nucleotide position.

### Paired-end *de novo* sequence assembly

Paired-end *de novo* assembly of the influenza genome was performed using Geneious version 5.0.1 software [54], with strict parameters (word length  = 18; 0% mismatch/gap tolerance; only paired-end reads included in assembly; expected paired-end mate distance = 200) to avoid misassembly, The *de novo* assembly algorithm used by Geneious is a greedy algorithm similar to that used in multiple sequence alignment programs such as ClustalW (Matt Kearse, Geneious Inc., personal communication) [55]. Initial generated contigs of size ≥100 bp were further assembled *de novo* in Geneious at high sensitivity with “fine tuning” of gaps and 3-nt trimming of contig ends. Final *de novo* assembled contigs were then mapped to the full-genome reference sequence A/California/06/2009(H1N1) (GenBank accession numbers FJ966960 – FJ966965 and FJ971074-FJ971075). Out of the remaining 561 contigs that did not map to influenza, ∼90% of the contigs corresponded to low-complexity sequence that was identified as such by applying an LZW compression ratio of 0.5, slightly higher than that used in the initial filtering (0.4).

## Supporting Information

Figure S1 Coverage map of the influenza genome for 2009 H1N1 samples from Canada. The coverage is plotted on a log scale as a function of nucleotide position.
(0.96 MB EPS)

Figure S2 Coverage map of the influenza genome for 2009 H1N1 samples from California and Mexico. The coverage is plotted on a log scale as a function of nucleotide position.
(0.92 MB EPS)

Table S1 Study patients with 2009 H1N1 influenza (n = 17).
(0.07 MB XLS)

Table S2 2009 H1N1 single nucleotide polymorphisms in samples Cal-UC2 and BC-22. Nonambiguous SNPs in samples Cal-UC2 and BC-22 were determined by alignment to the A/California/04/2009 (H1N1) reference genome. The nucleotide position is based on the A/California/04/2009 reference sequence.
(0.07 MB XLS)

Table S3 Primers used for whole-genome Sanger sequencing of 2009 H1N1 samples BC-22 and Cal-UC2.
(0.05 MB XLS)

## References

[1] Dawood FS, Jain S, Finelli L, Shaw MW, Novel Swine-Origin Influenza AVIT (2009). Emergence of a novel swine-origin influenza A (H1N1) virus in humans.. N Engl J Med.

[2] Shinde V, Bridges CB, Uyeki TM, Shu B, Balish A (2009). Triple-reassortant swine influenza A (H1) in humans in the United States, 2005-2009.. N Engl J Med.

[3] Fraser C, Donnelly CA, Cauchemez S, Hanage WP, Van Kerkhove MD (2009). Pandemic potential of a strain of influenza A (H1N1): early findings.. Science.

[4] Faix DJ, Sherman SS, Waterman SH (2009). Rapid-test sensitivity for novel swine-origin influenza A (H1N1) virus in humans.. N Engl J Med.

[5] Smith GJ, Vijaykrishna D, Bahl J, Lycett SJ, Worobey M (2009). Origins and evolutionary genomics of the 2009 swine-origin H1N1 influenza A epidemic.. Nature.

[6] Tang P, Chiu C (2010). Metagenomics for the discovery of novel human viruses.. Future Microbiol.

[7] Chiu CY, Rouskin S, Koshy A, Urisman A, Fischer K (2006). Microarray detection of human parainfluenzavirus 4 infection associated with respiratory failure in an immunocompetent adult.. Clin Infect Dis.

[8] Wang D, Coscoy L, Zylberberg M, Avila PC, Boushey HA (2002). Microarray-based detection and genotyping of viral pathogens.. Proc Natl Acad Sci U S A.

[9] Rota PA, Oberste MS, Monroe SS, Nix WA, Campagnoli R (2003). Characterization of a novel coronavirus associated with severe acute respiratory syndrome.. Science.

[10] Wang D, Urisman A, Liu YT, Springer M, Ksiazek TG (2003). Viral discovery and sequence recovery using DNA microarrays.. PLoS Biol.

[11] Kistler A, Avila PC, Rouskin S, Wang D, Ward T (2007). Pan-viral screening of respiratory tract infections in adults with and without asthma reveals unexpected human coronavirus and human rhinovirus diversity.. J Infect Dis.

[12] Chiu CY, Greninger AL, Kanada K, Kwok T, Fischer KF (2008). Identification of cardioviruses related to Theiler's murine encephalomyelitis virus in human infections.. Proc Natl Acad Sci U S A.

[13] Ganem D, Kistler A, Derisi J (2010). Achalasia and viral infection: new insights from veterinary medicine.. Sci Transl Med.

[14] Kistler AL, Gancz A, Clubb S, Skewes-Cox P, Fischer K (2008). Recovery of divergent avian bornaviruses from cases of proventricular dilatation disease: identification of a candidate etiologic agent.. Virol J.

[15] Chiu CY, Urisman A, Greenhow TL, Rouskin S, Yagi S (2008). Utility of DNA microarrays for detection of viruses in acute respiratory tract infections in children.. J Pediatr.

[16] Finkbeiner SR, Li Y, Ruone S, Conrardy C, Gregoricus N (2009). Identification of a novel astrovirus (astrovirus VA1) associated with an outbreak of acute gastroenteritis.. J Virol.

[17] Greninger AL, Runckel C, Chiu CY, Haggerty T, Parsonnet J (2009). The complete genome of klassevirus - a novel picornavirus in pediatric stool.. Virol J.

[18] Victoria JG, Kapoor A, Li L, Blinkova O, Slikas B (2009). Metagenomic analyses of viruses in stool samples from children with acute flaccid paralysis.. J Virol.

[19] Li L, Victoria JG, Wang C, Jones M, Fellers GM (2010). Bat guano virome: predominance of dietary viruses from insects and plants plus novel mammalian viruses.. J Virol.

[20] Li L, Kapoor A, Slikas B, Bamidele OS, Wang C (2010). Multiple diverse circoviruses infect farm animals and are commonly found in human and chimpanzee feces.. J Virol.

[21] Briese T, Paweska JT, McMullan LK, Hutchison SK, Street C (2009). Genetic detection and characterization of Lujo virus, a new hemorrhagic fever-associated arenavirus from southern Africa.. PLoS Pathog.

[22] Palacios G, Druce J, Du L, Tran T, Birch C (2008). A new arenavirus in a cluster of fatal transplant-associated diseases.. N Engl J Med.

[23] Treanor J (2004). Influenza vaccine–outmaneuvering antigenic shift and drift.. N Engl J Med.

[24] Lin B, Wang Z, Vora GJ, Thornton JA, Schnur JM (2006). Broad-spectrum respiratory tract pathogen identification using resequencing DNA microarrays.. Genome Res.

[25] Mehlmann M, Dawson ED, Townsend MB, Smagala JA, Moore CL (2006). Robust sequence selection method used to develop the FluChip diagnostic microarray for influenza virus.. J Clin Microbiol.

[26] Palacios G, Quan PL, Jabado OJ, Conlan S, Hirschberg DL (2007). Panmicrobial oligonucleotide array for diagnosis of infectious diseases.. Emerg Infect Dis.

[27] Townsend MB, Dawson ED, Mehlmann M, Smagala JA, Dankbar DM (2006). Experimental evaluation of the FluChip diagnostic microarray for influenza virus surveillance.. J Clin Microbiol.

[28] Lu Q, Zhang XQ, Pond SL, Reed S, Schooley RT (2009). Detection in 2009 of the swine origin influenza A (H1N1) virus by a subtyping microarray.. J Clin Microbiol.

[29] Lee CW, Koh CW, Chan YS, Aw PP, Loh KH (2010). Large-scale evolutionary surveillance of the 2009 H1N1 influenza A virus using resequencing arrays.. Nucleic Acids Res.

[30] Wang Z, Daum LT, Vora GJ, Metzgar D, Walter EA (2006). Identifying influenza viruses with resequencing microarrays.. Emerg Infect Dis.

[31] Deyde VM, Sheu TG, Trujillo AA, Okomo-Adhiambo M, Garten R (2009). Detection of molecular markers of drug resistance in the 2009 pandemic influenza A (H1N1) viruses using pyrosequencing.. Antimicrob Agents Chemother.

[32] Nakamura S, Yang CS, Sakon N, Ueda M, Tougan T (2009). Direct metagenomic detection of viral pathogens in nasal and fecal specimens using an unbiased high-throughput sequencing approach.. PLoS ONE.

[33] Kuroda M, Katano H, Nakajima N, Tobiume M, Ainai A (2010). Characterization of quasispecies of pandemic 2009 influenza A virus (A/H1N1/2009) by de novo sequencing using a next-generation DNA sequencer.. PLoS ONE.

[34] Louie JK, Acosta M, Jamieson DJ, Honein MA, California Pandemic Working G (2010). Severe 2009 H1N1 influenza in pregnant and postpartum women in California.. N Engl J Med.

[35] Urisman A, Fischer KF, Chiu CY, Kistler AL, Beck S (2005). E-Predict: a computational strategy for species identification based on observed DNA microarray hybridization patterns.. Genome Biol.

[36] Stewart F, Ottesen E, DeLong E (2010). Development and quantitative analyses of a universal rRNA-subtraction protocol for microbial metatranscriptomics.. ISME J.

[37] Group NHW, Peterson J, Garges S, Giovanni M, McInnes P (2009). The NIH Human Microbiome Project.. Genome Res.

[38] Fischer N, Schulz C, Stieler K, Hohn O, Lange C (2010). Xenotropic Murine Leukemia Virus-related Gammaretrovirus in Respiratory Tract.. Emerg Infect Dis.

[39] Urisman A, Molinaro RJ, Fischer N, Plummer SJ, Casey G (2006). Identification of a novel Gammaretrovirus in prostate tumors of patients homozygous for R462Q RNASEL variant.. PLoS Pathog.

[40] Lombardi VC, Ruscetti FW, Das Gupta J, Pfost MA, Hagen KS (2009). Detection of an infectious retrovirus, XMRV, in blood cells of patients with chronic fatigue syndrome.. Science.

[41] Samuel CE (2001). Antiviral actions of interferons.. Clin Microbiol Rev.

[42] Feng H, Shuda M, Chang Y, Moore PS (2008). Clonal integration of a polyomavirus in human Merkel cell carcinoma.. Science.

[43] Willner D, Furlan M, Haynes M, Schmieder R, Angly FE (2009). Metagenomic analysis of respiratory tract DNA viral communities in cystic fibrosis and non-cystic fibrosis individuals.. PLoS ONE.

[44] Palacios G, Hornig M, Cisterna D, Savji N, Bussetti AV (2009). Streptococcus pneumoniae coinfection is correlated with the severity of H1N1 pandemic influenza.. PLoS ONE.

[45] Zhang T, Breitbart M, Lee WH, Run JQ, Wei CL (2006). RNA viral community in human feces: prevalence of plant pathogenic viruses.. PLoS Biol.

[46] Dohm JC, Lottaz C, Borodina T, Himmelbauer H (2008). Substantial biases in ultra-short read data sets from high-throughput DNA sequencing.. Nucleic Acids Res.

[47] Hale BG, Steel J, Medina RA, Manicassamy B, Ye J (2010). Inefficient control of host gene expression by the 2009 pandemic H1N1 influenza A virus NS1 protein.. J Virol.

[48] Eid J, Fehr A, Gray J, Luong K, Lyle J (2009). Real-time DNA sequencing from single polymerase molecules.. Science.

[49] Martinez DA, Nelson MA (2010). The next generation becomes the now generation.. PLoS Genet.

[50] Sorber K, Chiu C, Webster D, Dimon M, Ruby JG (2008). The long march: a sample preparation technique that enhances contig length and coverage by high-throughput short-read sequencing.. PLoS ONE.

[51] Arnulf I, Lin L, Gadoth N, File J, Lecendreux M (2008). Kleine-Levin syndrome: a systematic study of 108 patients.. Ann Neurol.

[52] Mi H, Thomas P (2009). PANTHER pathway: an ontology-based pathway database coupled with data analysis tools.. Methods Mol Biol.

[53] Ning Z, Cox AJ, Mullikin JC (2001). SSAHA: a fast search method for large DNA databases.. Genome Res.

[54] Drummond A, Ashton B, Cheung M, Heled J, Kearse M (2009). Geneious v4.7.. http://www.geneious.com.

[55] Thompson JD, Gibson TJ, Higgins DG (2002). Multiple sequence alignment using ClustalW and ClustalX.. Curr Protoc Bioinformatics Chapter.

